# Epidermal growth factor receptor variant type III markedly accelerates angiogenesis and tumor growth via inducing c-myc mediated angiopoietin-like 4 expression in malignant glioma

**DOI:** 10.1186/1476-4598-12-31

**Published:** 2013-04-25

**Authors:** Yasufumi Katanasaka, Yasuo Kodera, Yuka Kitamura, Tatsuya Morimoto, Tomohide Tamura, Fumiaki Koizumi

**Affiliations:** 1 Shien-lab, National Cancer Center Hospital, 5-1-1 Tsukiji, Chuo-ku, Tokyo, Japan; 2 Division of Internal Medicine, National Cancer Center Hospital, 5-1-1 Tsukiji, Chuo-ku, Tokyo, Japan; 3 Division of Genetics, Genomics and Genetics Group, National Cancer Center Research Institute, 5-1-1 Tsukiji, Chuo-ku, Tokyo, Japan; 4 Division of Molecular Medicine, University of Shizuoka, 52-1 Yada, Shizuoka, Japan

**Keywords:** Malignant glioma, Angiogenesis, Epidermal growth factor variant type III, Angiopoietin -like 4, c-Myc

## Abstract

**Background:**

Expression of the constitutively activated mutant EGFR variant III (EGFRvIII), the most common mutation in glioblastoma multiforme (GBMs), has been clinically correlated with tumor proliferation, invasion, and angiogenesis. In this study, we examined the role of EGFRvIII on the tumor microenvironment, especially on angiogenesis.

**Methods:**

To study the role of EGFRvIII in tumor angiogenesis, we prepared LN229 glioblastoma transfected with enhanced green fluorescent protein (EGFP), wild-type EGFR, or EGFRvIII (LN229-WT or -vIII), and examined tumor growth and microvessel density in the tumors. Additionally, the potential angiogenic factors were identified by real-time PCR analysis, and the functions in LN229-vIII cells were examined.

**Results:**

LN229-vIII cells showed more aggressive tumor growth and higher vascularity as compared to LN229-WT cells in vivo, although there was no significant difference in the cell growth rates in vitro. We next investigated the expression of 60 angiogenesis-related factors to clarify the mechanisms underlying the difference in vascularity between tumor xenografts of LN229-vIII and LN229-WT. We found that the mRNA and protein expressions of angiopoietin-like 4 (Angptl4), a secreted protein involved in angiogenesis and metabolism regulation, were significantly induced by EGFRvIII overexpression, both in vitro and in vivo. Constitutive knockdown of Angptl4 in LN229-vIII using shRNA significantly decreased the microvessel density in the tumor xenografts and suppressed tumor growth. To clarify the regulatory mechanisms of Angptl4 by EGFRvIII, we analyzed the signaling pathways and transcription factors by pharmacological inhibition and RNA interference. U0126, an ERK signal inhibitor dramatically suppressed Angptl4 expression. The transcription factor c-Myc, which is regulated by ERK, was activated in the LN229-vIII cells and knockdown of c-Myc using siRNA also attenuated Angptl4 expression in the LN229-vIII cells. Furthermore, chromatin immunoprecipitation (ChIP) assay revealed increased recruitment of c-Myc to the promoter region of Angptl4 in the LN229-vIII cells.

**Conclusions:**

In summary, we demonstrated that EGFRvIII induces Angptl4 expression through the ERK/c-Myc pathway and promotes tumor angiogenesis in malignant gliomas.

## Background

Glioblastoma multiforme (GBM), classified as a grade IV astrocytoma, has an extremely poor prognosis [1]. Long-term survival of patients with malignant gliomas has not improved substantially despite the development of multimodality treatments, including cytoreductive surgery, adjuvant radiation therapy, and cytotoxic chemotherapy. In order to develop additional therapeutic strategies, further understanding of the molecular genetics, biology and immunology of gliomas is desired.

GBMs are distinguished pathologically from lower-grade anaplastic astrocytomas by the presence of necrosis and microvascular hyperplasia, a florid form of angiogenesis [2]. Above all, a striking feature of GBMs is the presence of increasing neovascularization [3]. Many studies have demonstrated that glioma growth is dependent on the generation of tumor-associated blood vessels [4,5], therefore, use of antiangiogenic strategies is considered as a promising approach for the treatment of malignant gliomas.

There has been important progress in the elucidation of the molecular pathogenesis of malignant gliomas. Two common and highly specific genetic events associated with the GBM histology are epidermal growth factor receptor (EGFR) amplification and loss of the phosphatase and tensin homologue on chromosome 10 (PTEN) [6,7]. Many studies have revealed that EGFR is functionally dysregulated in various tumors. Dysregulation of signal transduction processes affects a variety of downstream biological processes associated with gene transcription and protein translation, cell proliferation, migration, adhesion, invasion, and angiogenesis [8]. Abnormalities of EGFR signaling have also been reported to be observed frequently in GBMs [9]. EGFR gene amplification or overexpression is detected in approximately 40% of patients with these tumors [10,11].

The EGFR variant type III (EGFRvIII), the most common mutation of EGFR in GBMs, is reported to be present in 25% to 33% of all cases of GBMs, but only in those showing EGFR amplification and overexpression [12]. EGFRvIII overexpression has been shown to induce tumor growth of GBMs [13] and reported to be correlated with a poor prognosis in clinical settings [14,15]. This EGFR variant is the result of deletion of exons 2 to 7 including the extracellular ligand-binding domain, and its receptor tyrosine kinase is constitutively active [9]. Because it is not present in normal tissues, it is considered as a potential target for tumor-specific therapy. Currently, considerable effort is being made for the development of anti-EGFRvIII agents, such as vaccines and specific antibodies [7,16,17].

EGFR signaling promotes not only cell growth, but also angiogenesis by induction of proangiogenic factors such as the vascular endothelial growth factor (VEGF) and interleukin-8 (IL-8) [18]. Although the NF-kB/IL-8 pathway contributes to tumor angiogenesis in EGFRvIII-overexpressing glioblastomas [19], the EGFRvIII signaling pathways involved in the promotion of angiogenesis have not yet been clearly elucidated. In this study, we show the involvement of EGFRvIII in tumor angiogenesis in LN229, a GBM cell line, and that the induction of angiopoietin-like 4 (Angptl4) expression by c-Myc is involved in EGFRvIII-induced angiogenesis.

## Results

### Promotion of tumor angiogenesis by EGFRvIII overexpression

**Figure 1:**
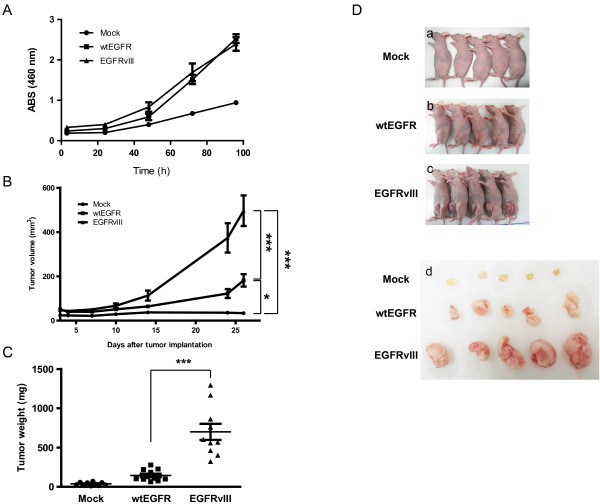
**EGFRvIII promotes tumor growth in a mouse xenograft model.** (**A**) The cells (2,000 cells/well) were incubated for 96 hours, and the cell viability was measured at the indicated times using WST-8. Data shown are the means ± SD (n = 5). (**B**) Tumor volume in the mice implanted with mock, wtEGFR, and EGFRvIII-transfected LN229 cells. LN229 cells (3.0 × 10^6^ cells/mouse) were subcutaneously implanted into BALB/c nu/nu female mice. (**C**) The tumors were extracted from the mice and weighed at day 26 after tumor implantation. (**D**) The images of the mice implanted with tumors (**a**-**c**) and of the extracted tumors are shown (**d**). Data shown are the means ± SEM (n = 10). Significant differences are shown: * *p*<0.05, *** *p*<0.001.

To examine the involvement of EGFRvIII in angiogenesis, LN229 glioblastoma cells were transduced with retrovirus vectors encoding enhanced green fluorescent protein (mock), wild-type EGFR (wtEGFR), or EGFRvIII. The transfected cells were sorted by EGFP expression from the viral expression vector using flow cytometry. We observed that most of the cells expressed EGFP and were altered morphologically (Additional file 1: Figure S1A), and also confirmed the expression of wtEGFR and EGFRvIII by RT-PCR and western blotting (Additional file 1: Figure S1B and C). The methods of additional figures described in an additional document (Additional file 2: Supplementary methods). The cell growth ratio and migration of mock, wtEGFR-, or EGFRvIII-overexpressing LN229 (LN229-WT or LN229-vIII) cells were examined in vitro. No significant change in cell growth rate was observed (Figure 1A) and cell migration was significantly increased in LN229-vIII (Additional file 1: Figure S1D). We then examined the effect of wtEGFR and EGFRvIII on tumor growth in vivo. Tumor growth was significantly enhanced in the mice bearing tumor xenografts of LN229-vIII as compared with that in the mice bearing tumor xenografts of LN229-WT (Figure 1B-D), as previously reported [13,20].

**Figure 2:**
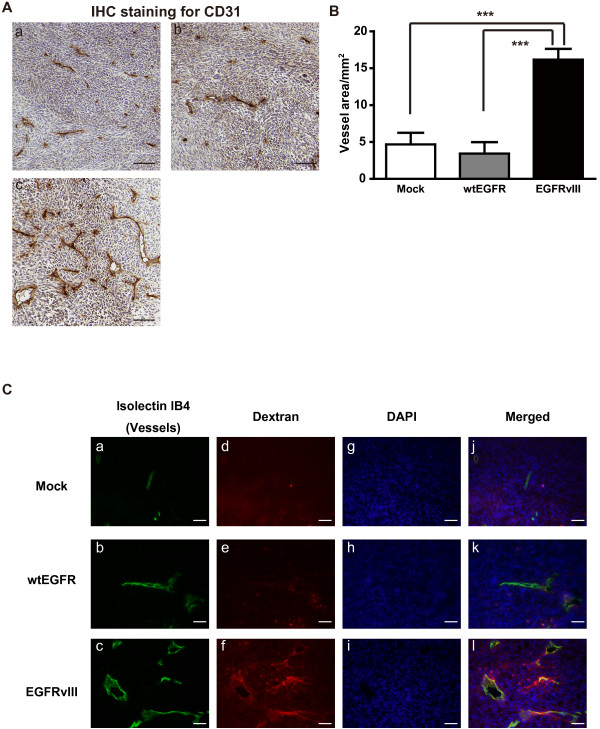
**EGFRvIII increases the microvessel density and vascular permeability in tumors.** (**A**) Frozen sections of tumor tissues extracted from the mice implanted with LN229 cells transfected with mock (**a**), wtEGFR (**b**), EGFRvIII (**c**) were prepared and immunohistochemically stained for CD31, an endothelial cell marker. Scale bars show 100 μm. (**B**) Quantitative analysis of the microvessel density in the tumors was performed. At least three fields were selected from each section. Vessel area/mm^2^ was measured as the average of the values in four sections/mouse. Data shown are the means ± SEM (n = 5 mice). Significant differences are shown: *** *p*<0.001. (**C**) Permeability of the tumor blood vessels in LN229-tumor bearing mice. The mice were intravenously injected with TexasRed conjugated dextran (Mw. 70,000, 25 mg/mouse, red, **d**-**f**). Subsequently, 6 h after the dextran injection, Alexa647-conjugated isolectin IB4 (green, **a**-**c**), a probe used for specific labeling of endothelial cells, was administered. The mice were sacrificed, and frozen sections were prepared and observed by fluorescent microscopy. The cell nucleus were labeled with DAPI (blue, **g**-**i**). The merged images are shown (**j**-**l**). Scale bars show 50 μm.

We hypothesized that the microenvironment in the tumors was altered and was involved in the significant tumor progression, and investigated whether EGFRvIII also promoted tumor angiogenesis in vivo. Frozen sections of the tumors were prepared and immunostained for CD31, a representative endothelial cell marker, to examine the microvessel density in the tumors. The microvessel density (vessel area/mm^2^) was significantly augmented in the EGFRvIII-overexpressing tumors as compared with that in the mock- and wtEGFR-expressing tumors (Figure 2A and B). Since the tumor vasculature is a loose structure and highly permeable [21], we investigated the vascular permeability in the EGFRvIII-overexpressing tumors. Dextran is a macromolecule that leaks from hyperpermeable blood vessels [22]. Significant increase in the leakage of fluorescent-labeled dextran from the blood vessels was observed in the EGFRvIII-overexpressing tumors at 6 h after its administration, in contrast to the findings in the mock- and wtEGFR-expressing tumors (Figure 2C). These data suggest that EGFRvIII increases the vascular permeability as well as the microvessel density.

### Real-time PCR analysis for identification of EGFRvIII-related angiogenic factors

**Figure 3:**
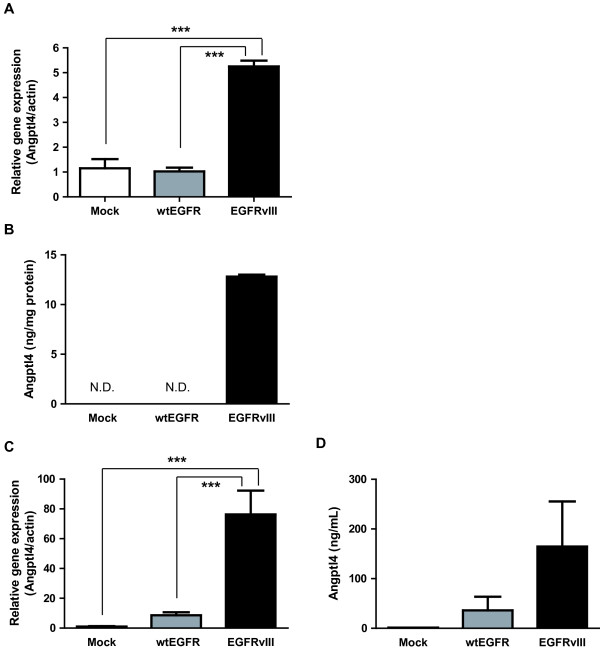
**EGFRvIII induces the expression of angiopoietin-like 4 both in vitro and in vivo.** Real-time PCR assay of samples obtained from mock, wtEGFR, and EGFRvIII-transfected LN229 cells (**A**) and tumors (**C**) was performed. Total RNA was extracted from the cells or excised tumors and reverse-transcribed. Real-time PCR was performed based on TaqMan Gene Expression Assays. Beta-actin was used as the endogenous control. ELISA was performed with cell-conditioned medium (**B**) and the supernatants of the homogenized tumors (**D**). Data shown are the means ± SEM (n=4-6). Significant differences are shown: *** *p*<0.001. N.D. denotes not detected.

Tumor angiogenesis is caused by a disruption of the balance between proangiogenic and antiangiogenic factors [23]. Since EGFRvIII increased both the microvessel density and vascular permeability in the tumor xenografts, it is likely that it also alters the expression and secretion of angiogenic factors. To investigate the angiogenic factors regulated by EGFRvIII, we analyzed the mRNA expressions of these factors by real-time PCR using a TaqMan Array Gene Signature 96-Well Plate for Angiogenesis. The analysis showed differences in the mRNA expressions of ANGPTL4, SERPINB5, KIT, FOXC2, COL15A1, F2, THBS2 and ITGB3 in the LN229-vIII cells as compared with that in the mock- and LN229-WT cells (Additional file 3: Table S1). Among these, the expression of Angptl4, which has been reported to be a secreted protein with proangiogenic activity [24], was markedly upregulated by EGFRvIII overexpression. Therefore, we focused on this protein and examined its expression at the mRNA and protein levels both in vitro and in vivo. Increase in Angptl4 expression was confirmed by both real-time PCR and ELISA in vitro (Figure 3A and B). Moreover, increase of Angptl4 expression in the mice bearing tumor xenografts of LN229-vIII was observed at both the mRNA and protein levels (Figure 3C and D). In our experiments, while the Angptl4 protein was detected in all EGFRvIII-overexpressing tumors, it was detected in only one of five mock- and two of five wtEGFR-expressing tumors.

### Knockdown of Angptl4 suppressed the growth of EGFRvIII-overexpressing tumors and tumor angiogenesis

**Figure 4:**
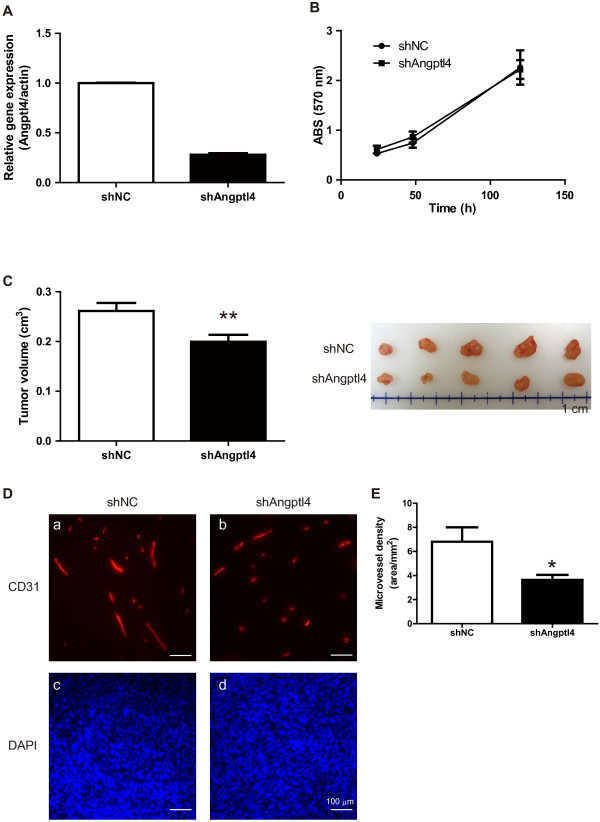
**Knockdown of Angptl4 suppresses tumor growth and angiogenesis.** (**A**) The LN229vIII cells were transfected with a retrovirus vector encoding negative control shRNA (shNC) or Angptl4-targeted shRNA (shAngptl4). The knockdown efficiency of Angptl4 in the LN229vIII cells was examined by real-time PCR analysis. (**B**) Cell growth of LN229vIII transfected with shNC or shAngptl4 was measured using WST-8. (**C**) Balb/c nu/nu mice were implanted subcutaneously with LN229vIII shNC or shAngptl4 cells, and the tumor volumes were measured on day 21. The excised tumors were photographed. (**D**) Frozen sections were prepared from the extracted tumors and immunostained for CD31, an endothelial cell marker (**a** and **b**). The nucleus was stained with DAPI (**c** and **d**). Scale bars show 100 μm. (**E**) Quantitative analysis of the microvessel density in the tumors was performed. At least three fields were selected from each section. Vessel area/mm^2^ was measured as the average of the values in four sections/mouse. Data shown are the means ± SEM (n=5 mice). Significant differences are shown: * *p*<0.05, ** *p*<0.01.

To clarify the role of Angptl4 in the growth and angiogenesis in tumors formed by LN229-vIII cells, we prepared cells with constitutive knockdown of Angptl4. We designed short hairpin RNA (shRNA) to perform knockdown of Angptl4 with shRNA-expressed retrovirus vector. After the virus infection and culturing of cells in G418-containing media, the mRNA expression of Angptl4 was significantly decreased in LN229-vIII cells as measured by real time PCR analysis (Figure 4A), while the growth ratio of the cells was not significantly altered (Figure 4B). The cells expressing shRNA for negative control (shNC) or Angptl4 (shAngptl4) were subcutaneously implanted into mice. The tumor volume at day 14 after implantation of the cells was significantly suppressed by shAngptl4 (Figure 4C). Tumor sections were prepared for examination of the microvessel density; the microvessel density was significantly decreased in tumor xenografts of the Angptl4-knockdown cells (Figure 4D). These results suggest that Angplt4 promotes, at least in part, tumor angiogenesis in EGFRvIII-overexpressing tumors.

### Transcriptional regulation of Angptl4 by c-Myc

**Figure 5:**
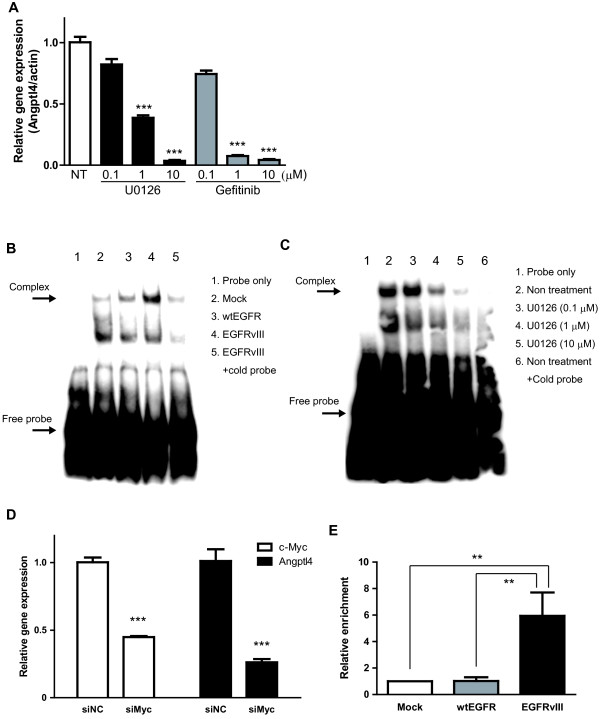
**Angptl4 gene transcription is mediated by the ERK/c-Myc pathway in LN229-vIII cells.** (**A**) Inhibition of Angptl4 mRNA expression by treatment with U0126. LN229-vIII cells were treated with U0126 or gefitinib for 24 h. Total RNA was extracted and reverse- transcribed, followed by real-time PCR. (**B**) EMSA assay for Myc/MAX using a nuclear extract of LN229 cells transfected with mock, wtEGFR and EGFRvIII. (**C**) LN229-vIII cells were treated with U0126 (0.1, 1, 10 μM) for 24 h; thereafter, the nuclear proteins were extracted and used in the EMSA assay. Complex, the specific DNA-protein (probe/c-Myc) complex. (**D**) Knockdown of c-Myc in the LN229-vIII cells was accomplished using siRNA. Angptl4 (black bar) and c-Myc (white bar) mRNA expressions were determined by real-time PCR and normalized to the expression level of β-actin. Bars show the mean ± SEM (n=4). (**E**) Formalin-fixed chromatin-protein complexes were sonicated, immunoprecipitated for c-Myc, eluted, and quantitative PCR for the promoter of Angptl4 was performed. The enrichment was calculated in fold-values by normalization to the negative control IgG and input control. Bars show the means ± SEM (n=3). Significant differences are shown: ** *p*<0.01, *** *p*<0.001.

Although it has been reported that Angptl4 transcription is regulated by the MAPK signal cascade [25], the involvement of Angptl4 transcription in EGFR signaling in glioma cells is largely unknown. EGFR alters the transcriptional regulation of many molecules via various signaling pathways. We therefore investigated the transcriptional regulation of Angptl4 expression by using inhibitors of signaling pathways including MEK/ERK, JNK, p38, PI3K/Akt, and JAK which are known to be downstream of the phosphorylation of EGFR [26-29]. Among these, U0126 treatment dramatically decreased Angptl4 expression in the LN229-vIII cells (Figure 5A and Additional file 4: Figure S2). In addition, PD98059 (MEK1 inhibitor) and FR180204 (ATP competitor of ERK1/2) also decreased Angptl4 mRNA expression in the cells (Additional file 4: Figure S2). We next investigated which transcription factors might contribute to the Angptl4 mRNA expression in LN229-vIII cells. A transcription factor database search analysis revealed that the promoter of Angptl4 includes a consensus sequence for c-Myc/Max. The activity of the transcription factor c-Myc is regulated by various signaling molecules, such as ERK [30]. We therefore hypothesized that c-Myc be activated in LN229-vIII cells through MAPK signaling to promote Angptl4 transcription. We then investigated the transcriptional regulation of Angptl4 by c-Myc. A gel-shift assay showed that Myc/Max was activated in the LN229-vIII cells and that the activation was suppressed by treatment with U0126 (Figure 5B and C). To clarify the role of c-Myc in Angptl4 transcription, an experiment using RNAi against c-Myc was also performed. Angptl4 mRNA expression in the LN229-vIII cells was significantly decreased by the knockdown of c-Myc using siRNA (Figure 5D). Similar results were obtained using another siRNA for c-Myc (data not shown). In a ChIP assay, binding of c-Myc to the promoter sequence on Angptl4 was detected and the binding was significantly enhanced in the LN229-vIII cells (Figure 5E). These findings indicate that c-Myc is activated through the MAPK pathway in the LN229-vIII cells to directly regulate Angptl4 transcription.

## Discussion

Although EGFRvIII has been shown to promote tumor growth of gliomas through various signaling pathways [9], the key signal molecules involved in the alteration of the tumor microenvironment have not yet been fully elucidated. In this study, we investigated whether EGFRvIII contributes to tumor angiogenesis, and showed dramatic increases in the microvessel density and vascular permeability in tumor xenografts of LN229-vIII as compared to LN229-WT in mice, consistent with the results of a previous study [19]. Considering that hypervascularity is a distinctive pathological characteristic of malignant gliomas [31,32], the EGFRvIII expression status may have a great impact on the clinical picture. Although EGFR is known to promote angiogenesis by induction of proangiogenic factors, such as VEGF-A and interleukin 8 [18,33], no dramatic induction of angiogenesis by wtEGFR was observed in our experiments. This difference leads to the speculation that constitutive activation of EGFR may trigger striking induction of various transcripts, including pro-angiogenic factors. In order to examine the molecular mechanisms underlying the induction of angiogenesis by EGFRvIII, the expressions of 60 angiogenic factors in LN229 cells were examined by real-time PCR analysis. Although VEGF-A is a representative angiogenic factor and a possible therapeutic target for glioblastoma [34], VEGF-A induction by EGFRvIII was observed only to a certain extent *in vivo* (data not shown), and not at all *in vitro* (Additional file 3: Table S1). Among the 60 angiogenic factors, we first found that Angptl4 expression was significantly induced by EGFRvIII overexpression, and that Angptl4 acts as a pro-angiogenic factor in tumor xenografts. Recently, Bonavia, et al. showed that the NF-kB/IL-8 pathway plays important roles in EGFRvIII-induced angiogenesis and growth in gliomas [19], however, no significant change of the IL-8 expression was observed in our in vitro experiment (Additional file 3: Table S1). It is likely that the differences between our results and those of the previous report are related to differences in the cell lines.

The molecular mechanisms of Angptl4-induced angiogenesis in malignant gliomas still remain largely unknown. Angptl4 is expressed in the liver, adipose tissue and placenta, as also in ischemic tissues [35]. It is a member of the angiopoietin family and is a target of members of the peroxisome proliferator-activated receptor (PPAR) family, which are known as metabolic-response transcription factors [36]. It has been reported that expression of Angptl4 is upregulated under various conditions including hypoxia and caloric restriction, and transcription factors such as PPARγ and Smad have been shown to regulate its expression [35,37,38]. Increased Angptl4 expression has been shown in a variety of tumor tissues, such as oral Kaposi’s sarcoma, esophageal squamous cell carcinoma, gastric cancer, and colorectal cancer [39-42]. Since a number of reports have indicated the effects of Angptl4 on angiogenesis, including endothelial cell proliferation, migration, differentiation, endothelial cell adhesion, and vascular permeability [43-46], it seems likely that Angptl4 contributes to the increased angiogenesis and vascular permeability in gliomas formed by EGFRvIII cells. Moreover, it has been demonstrated that Angptl4 disrupts vascular endothelial cell-cell junctions and promotes lung metastasis of breast cancer cells expressing transforming growth factor-β [35], while preventing metastasis of melanoma cells [47] and also inhibiting angiogenesis [48]. These diverse and often conflicting results suggest that Angptl4 exhibit tissue-specific activity and act in accordance with the prevailing cellular environment.

Our results suggest that Angptl4 transcription is regulated, at least partially, by EGFRvIII/ERK/c-Myc-mediated signaling. EGFR activation induces Ras/MEK/ERK phosphorylation, and phosphorylated ERK activates various transcription factors. It has been shown that MAPK signaling contributes to Angptl4 expression [25]. Myc is known as an ERK-activated transcription factor [30]. Wild-type EGFR expression, as compared to mock, increased tumor growth and Angptl4 expression *in vivo*, and also activated ERK phosphorylation in the LN229 cells; however, the degree of activation was not significantly different from that induced by EGFRvIII expression (data not shown). These data suggest that, although the MAPK pathway plays an important role in c-Myc activation, other factors are also involved in the marked activation of c-Myc and induction of Angptl4 expression in the LN229-vIII cells. The promoter region of Angptl4 contains the consensus sequence of c-Myc, ‘CACGTG’. The results of the ChIP assay revealed enhanced binding between c-Myc and the promoter region of Angptl4 in LN229-vIII cells, suggesting that the transcriptional regulation of Angptl4 by c-Myc might contribute to the induction of angiogenesis in gliomas. An MEK inhibitor was also found to markedly inhibit Angptl4 expression in EGFRvIII-overexpressing LN229 cells. In a previously reported study, combined use of an MEK inhibitor with a PI3K inhibitor effectively suppressed the growth of gliomas [49]. MEK inhibitors have been examined in clinical trials for various cancers, and their potential usefulness in the treatment of gliomas has been suggested.

## Conclusions

In conclusion, we demonstrated in this study that EGFRvIII induces Angptl4 expression through the ERK/c-Myc pathway, and that Angptl4 is a possible inducer of tumor angiogenesis in gliomas expressing EGFRvIII. Since EGFRvIII strongly induces neovascularization in the tumors, expression of EGFRvIII or Angptl4 may be a possible biomarker for predicting the effectiveness of antiangiogenic therapy, as well as serve as a therapeutic target, although further studies are needed.

## Methods

### Cell culture

The human glioblastoma cell lines LN229 (American Tissue Culture Collection) were maintained in Dulbecco’s minimal essential medium (DMEM, Sigma) supplemented with streptomycin (100 μg/ml), penicillin (100 units/mL), and 10% heat-inactivated fetal bovine serum (FBS) at 37°C under 5% CO_2_ in a humidified chamber. The cDNA for wild-type EGFR or EGFRvIII was transfected into LN229 cells by a retrovirus vector, as described previously [17], and the transfected cells were selected by GFP expression from the viral expression vector using a cell sorter (BD Biosciences).

### Cell proliferation assay

LN229 cells (1,000 cells/well) were seeded into a 96-well microtiter plate. After incubation for 24-96 h at 37ºC, the cell viability was measured with a Cell Counting Kit-8 (Dojindo, Tokyo, Japan) in accordance with the manufacturer’s instructions.

### RNA isolation, reverse-transcription PCR, and real-time PCR

Total RNA was isolated using Isogen (Nippon Gene, Tokyo, Japan) and the resulting RNA was reverse-transcribed with the High Capacity cDNA Reverse Transcription Kit (Applied Biosystems, Tokyo, Japan). Real-time PCR assay was performed on a StepOnePlus (Applied Biosystems) using the TaqMan Gene Expression Assays or a TaqMan Array Gene Signature 96-Well Plate (Angiogenesis, human, Applied Biosystems). The relative real-time PCR quantification was based on a comparative quantitation method.

### Western blotting

Western blotting was performed as described previously [50], with some modifications. The cells were washed with ice-cold PBS and lysed with M-PER (PIERCE, Tokyo, Japan) containing protease and phosphatase inhibitors. The protein concentration was determined using a BCA protein assay kit (PIERCE). The protein samples were mixed with SDS-PAGE sample buffer (2% SDS, 10% glycerol, 6% 2-mercaptoethanol, 50 mM Tris-HCl; pH 6.8), and an equal amount of proteins in each sample was subjected to SDS-PAGE. The separated proteins were transferred to a PVDF membrane (Millipore, Bedford, MA) and blocked with 5% skim-milk in TBST (0.9% NaCl, 0.1% Tween20, 20 mM Tris-HCl; pH7.4). The primary antibodies used were anti-EGFR antibody (BD Pharmingen, NJ) and anti-actin antibody (Sigma). Horseradish peroxidase (HRP)-conjugated antibodies (Cell Signaling Technology, Tokyo, Japan) were used as the secondary antibodies. The PVDF membrane was developed with the ECL reagent (GE Healthcare, Buckinghamshire, UK).

### Tumor xenograft model

LN229 cells were subcutaneously implanted (3.0 × 10^6^ cells/mouse) into the posterior flanks of 4-week old female BALB/c nu/nu mice. The tumor sizes were monitored as described previously [51]. Animal studies were carried out according to the Guideline for Animal Experiments, drawn up by the Committee for Ethics in Animal Experimentation of the National Cancer Center, which meet the ethical standards required by law and the guidelines about experimental animals in Japan.

### Microvessel density analysis

After tumor implantation, the mice were sacrificed under diethyl ether anesthesia, and the tumors were dissected and weighed. Immunostaining was performed as described previously [52]. The tumor tissues were embedded and frozen with dry ice/ethanol. Tumor frozen sections (7 μm) were prepared and air-dried for at least 1 h. The sections were fixed with cold acetone, blocked in goat serum for 10 min at room temperature, and then incubated with anti-mouse CD31 rat monoclonal antibody (BD Pharmingen) for 18 h at 4°C. The sections were then stained with ABC Elute kit, or anti-rat IgG-Alexa fluor 555 conjugates (Molecular Probes, Inc.) for immunohistochemistry and immunofluorescent staining, respectively. After mounting the sections, the images were examined and scanned with Biozero (Keyence, Tokyo, Japan) at 20 × magnification. For quantitative analysis, the vascular area/mm^2^ in the tumors was quantified by counting the CD31-positive area in independent hotspots of at least four different microscopic fields in each of five mice/group, using the ImageJ software. The four fields were averaged in each tumor and the averages for each animal used to express the final count ± SEM.

### Vascular permeability

The in vivo vascular permeability assay was performed as described previously with some modifications [53]. The tumor-implanted mice were intravenously injected with TexasRed conjugated dextran (50 mg/kg/mouse, Mw 70,000, Molecular probes, Inc). At 6 h after the injection, Alexa647-conjugated Isolectin IB4 (Molecular probes, Inc) was injected for fluorescent staining of the blood vessels. After 10 minutes, perfusion fixation was performed under ether anesthesia and the tumors were extracted from the mice. The extracted tumors were frozen and sectioned as described above. The sections were fixed with 4% paraformaldehyde, mounted, and observed by fluorescent microscopy as described above.

### Enzyme-linked immunosorbent assay (ELISA)

LN229 cells were seeded (3.0 × 10^5^ cells) in a 35-mm dish and incubated overnight. The medium was refreshed and the culture dish was incubated for a further 48 h at 37°C. The culture medium was collected and centrifuged at 1,000 g for 10 min. The supernatant was recovered and ELISA for Angptl4 was performed using the Human Angiopoietin-like 4 DuoSet ELISA kit (R&D Systems, Minneapolis, MN) with a sensitivity of 1.25 ng⁄mL, an intra-assay coefficient of variation of 0.6–7.6%, and an inter-assay coefficient of variation of 8.5–11.2%. The assay was performed in accordance with the manufacturer’s instructions. The remaining cells on the dishes were lysed and the amount of protein was measured by a BCA protein assay. Tumor tissues extracted from the mice were homogenized in PBS (–) and centrifuged at 10,000 × g for 10 min at 4°C. The supernatant was collected and ELISA was performed as described above. Duplicate measurements were performed in a single experiment.

### Electrophoretic mobility shift assay (EMSA)

Nuclear fractions were extracted from the LN229 cells using a Nuclear Extraction kit (Panomics, Redwood City, CA). The EMSA binding assay was carried out using a Panomics EMSA “gel shift” kit in accordance with the manufacturer’s instructions. Assays were conducted using a biotin-labeled double-stranded oligonucleotide having a consensus recognition sequence for Myc/Max purchased from Panomics. Protein-DNA complexes were separated using nondenaturing PAGE. The oligonucleotides were secondarily probed with HRP-conjugated streptavidin and developed with the component solution by LAS4000.

### RNAi experiment

The RNAi experiment was performed with the Lipofectamine RNAiMAX reagent (Invitrogen, Tokyo, Japan) in accordance with the manufacturer’s instructions. The sequences of siRNA for c-Myc were 5′-AGA CCU UCA UCA AAA ACA UTT-3′ (sense) and 5′-AUG UUU UUG AUG AAG GUC UCG-3′ (antisense), which were designed by Ambion, and the non-silencing control siRNA was purchased from Invitrogen. After incubation with the siRNA for 48 h at 37°C, the mRNA expressions of c-Myc and Angptl4 were quantitatively determined by real-time PCR. Short hairpin RNA targeting the Angptl4-including entry vector was designed and prepared by Invitrogen. The shRNA was subcloned to a retrovirus vector and used in the experiments as described in a previous study [17].

### ChIP assay

The ChIP assay was performed using the ChIP IT Express kit (Active Motif, Carlsbad, CA), in accordance with the manufacturer’s instructions. LN229 cells were fixed with 1% formaldehyde for 10 min. The cells were then washed, lysed, and sonicated to reduce DNA lengths to the range of 200 to 1500 bp. The chromatin/DNA complexes were incubated with antibodies to c-Myc (Santa Cruz Biotechnology, CA) or IgG (Cell Signaling Technology) overnight at 4°C. The immune complexes were precipitated, eluted, reverse-crosslinked, and treated with proteinase K. After extraction of the DNA fragments, real-time PCR analysis was performed using Power SYBR green PCR master mixes (Applied Biosystems). The primer for the promoter of Angptl4 was purchased from BioScience (Fredrick, MD), and was as follows: forward, 5′-TAC TAG CGG TTT TAC GGG CG-3′; reverse, 5′-TCG AAC AGG AGG AGC AGA GAG CGA-3′. The predicted PCR product included a c-Myc binding sequence. Relative enrichment was comparatively calculated using IgG negative control as described in eBioScience instructions.

### Statistical analysis

Significant differences were analyzed by an unpaired Student’s *t*-test or analysis of variance (ANOVA) with Tukey’s *post-hoc* test using the GraphPad Prism software (Version 5.0). *p* < 0.05 was considered to indicate statistically significant difference.

## Competing interests

The authors declare that they have no competing interests.

## Authors’ contributions

YK and FK conceived the idea, designed the experiments, and drafted the manuscript. YK, YK and YK performed the experiments. FK, TM and TT edited the manuscript. All authors read and approved the manuscript.

## Supplementary Material

Additional file 1: Figure S1 Validation of wtEGFR and EGFRvIII overexpression in LN229 cells (**A**-**C**). EGFRvIII promotes cellular migration in vitro (**D**). Click here for file

Additional file 2 Supplementary methods. Click here for file

Additional file 3: Table S1 Real time PCR analysis of angiogenesis-related genes in LN229-vIII cells. Click here for file

Additional file 4: Figure S2 nhibition of Angptl4 mRNA expression by treatment with MAPK signal inhibitors in EGFRvIII-overexpressing LN229 cells. Click here for file
