# Supplementary figures and images for: Epidermal growth factor receptor variant type III markedly accelerates angiogenesis and tumor growth via inducing c-myc mediated angiopoietin-like 4 expression in malignant glioma
Source: Mol Cancer. 2013 Apr 25;12:31. doi: 10.1186/1476-4598-12-31 (PMC3641008; doi:10.1186/1476-4598-12-31)

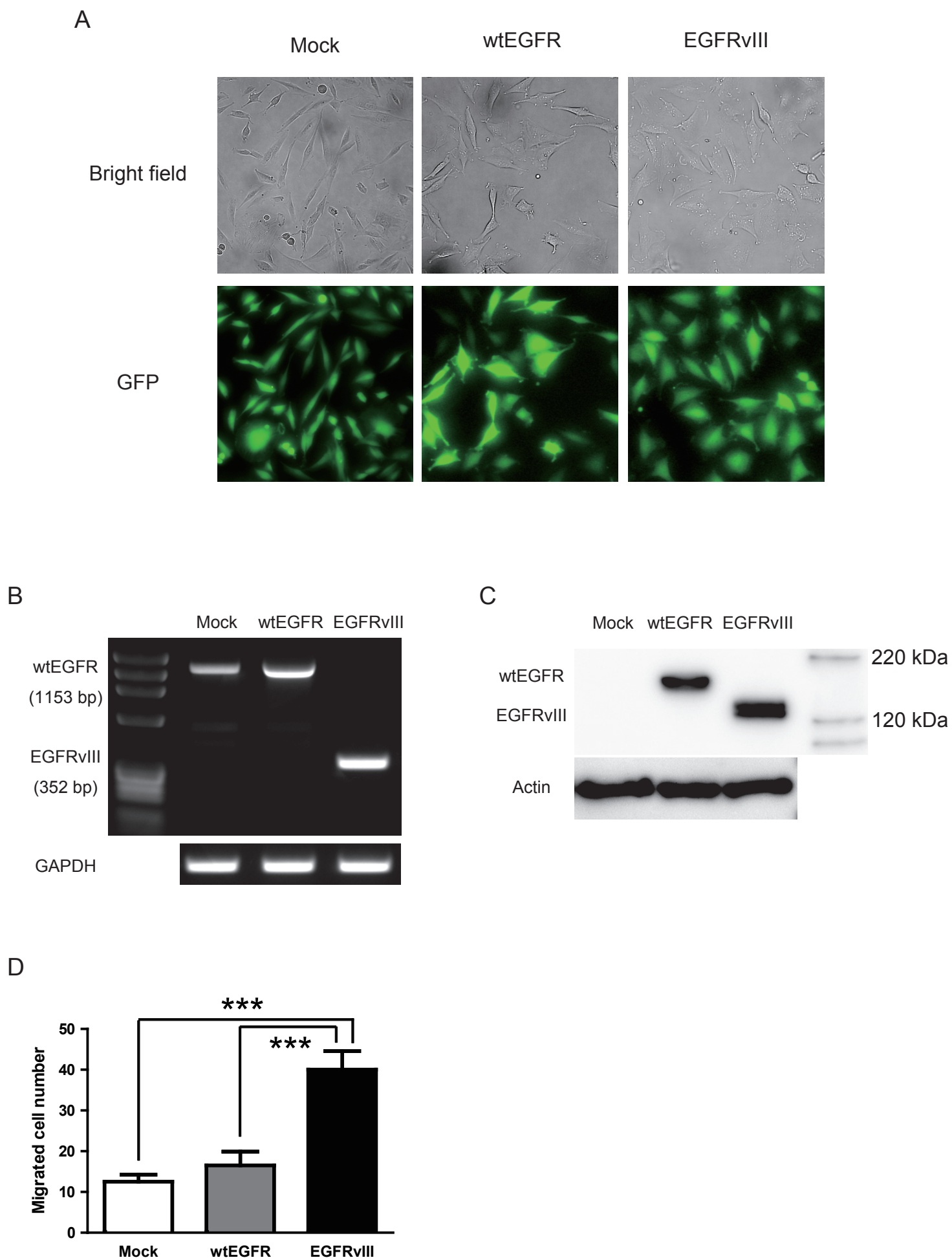

Supplement: Additional file 1: Figure S1 — Validation of wtEGFR and EGFRvIII overexpression in LN229 cells (A-C). EGFRvIII promotes cellular migration in vitro (D). [file 1476-4598-12-31-S1.pdf]

A

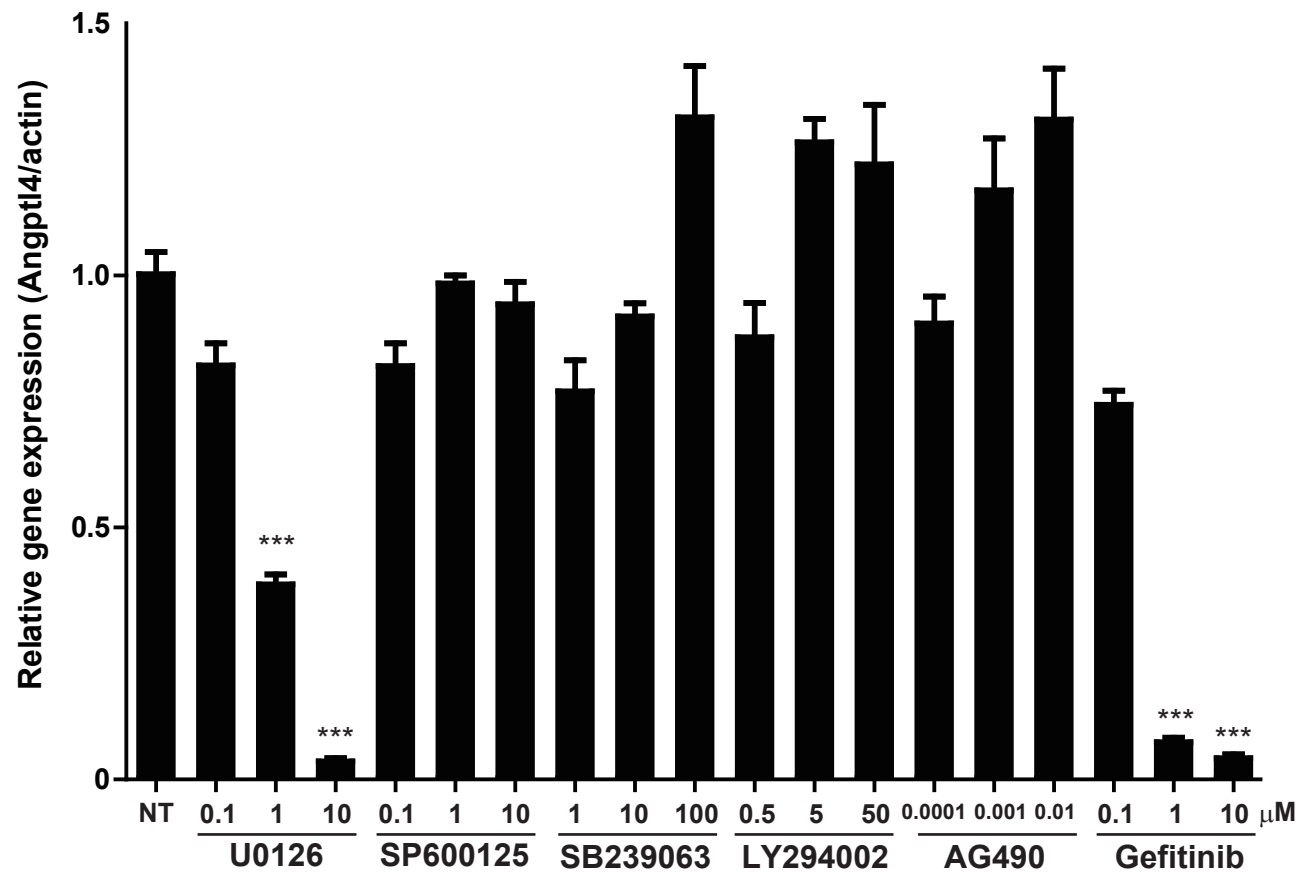

B

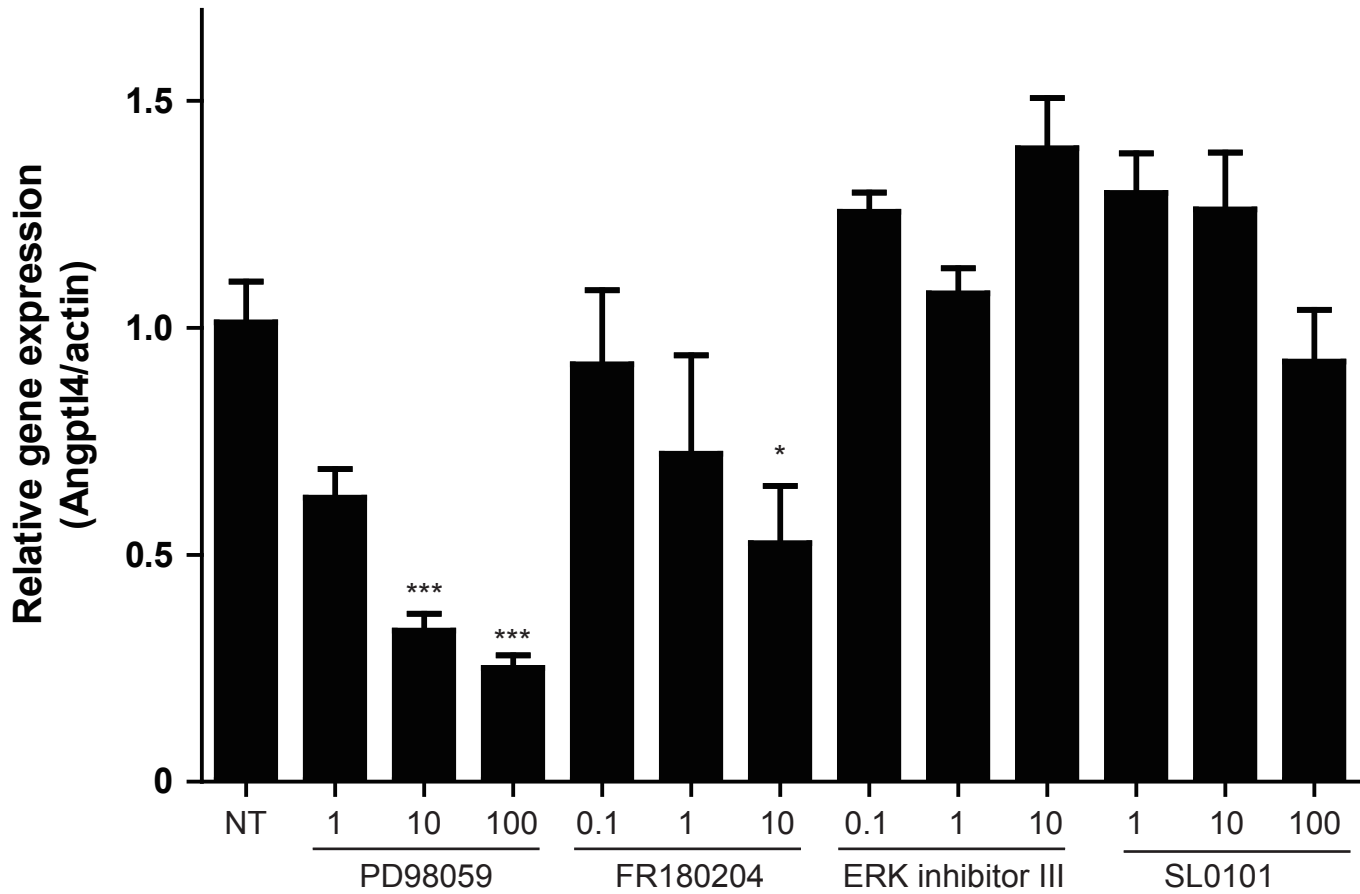

Supplement: Additional file 4: Figure S2 — nhibition of Angptl4 mRNA expression by treatment with MAPK signal inhibitors in EGFRvIII-overexpressing LN229 cells. [file 1476-4598-12-31-S4.pdf]
