# Supplementary material for: Epidermal growth factor receptor variant type III markedly accelerates angiogenesis and tumor growth via inducing c-myc mediated angiopoietin-like 4 expression in malignant glioma
Source: Mol Cancer. 2013 Apr 25;12:31. doi: 10.1186/1476-4598-12-31 (PMC3641008; doi:10.1186/1476-4598-12-31)
Supplement: Additional file 2 — Supplementary methods. [file 1476-4598-12-31-S2.pdf]

Supplementary methods

Materials and Methods

Materials

U0126, PD98059, FR180204, SL0101, ERK inhibitor III, and AG490 were purchased from Calbiochem (San Diego, CA). SB239063, LY294002, and SP600125 were purchased from SIGMA (Tokyo, Japan).

RNA isolation, reverse-transcription PCR, and real-time PCR

Total RNA was isolated using Isogen (Nippon Gene, Tokyo, Japan) and the resulting RNA was reverse-transcribed with High Capacity cDNA Reverse Transcription Kit (Applied Biosystems, Tokyo, Japan). PCR for the detection of wtEGFR (1153 bp) and EGFRvIII (352 bp) was performed using appropriate primer sets, as described previously [1]: 5'- ATG CGA CCC TCC GGG ACG - 3' and 5'- GAG TAT GTG TGA AGG AGT - 3'. The reaction was run under the following cycle conditions: 3 min denaturation at 94°C, followed by 35 cycles of 94°C for 45 s, 60°C for 45 s, and 72°C for 2 min, followed by final incubation at 72°C for 7 min. The RT-PCR products were resolved on a 1.5% agarose gel, stained with ethidium bromide (Molecular Probes Inc., Eugene, OR, USA), and visualized using LAS4000 (Fujifilm, Tokyo, Japan). Real-time

PCR assay was performed on a StepOnePlus (Applied Biosystems) using TaqMan Gene Expression Assays or TaqMan Array Gene Signature 96-Well Plate (Angiogenesis, human, Applied Biosystems). The relative real-time PCR quantification was based on a comparative quantitation method.

Cell migration assay

Migration assay were performed as described previously with some modification [2]. Briefly, LN229 cells in logarithmic growth phase were trypsinized and suspended in serum-free media at densities of 50,000 cells/mL. FBS (10%) was added to the lower chamber to serve as a chemoattractant. After 6 hours of incubation in a 37°C incubator, the cells that had migrated to the lower wells were observed fluorescently with Biozero (Keyence, Tokyo, Japan) and counted the cell number.

Figure S1

Validation of wtEGFR and EGFRvIII overexpression in LN229 cells.

(A) LN229 cells were infected with a retroviral vector expressing GFP and wtEGFR or EGFRvIII. Images were taken by fluorescent microscopy. (B) Total RNA was isolated from these transfected cells and reverse-transcribed. Then, PCR was performed to analyze the expression of wtEGFR (1153 bp) and EGFRvIII (352 bp). (C) The LN229

cells were lysed and an equal amount of protein was loaded onto SDS-PAGE. Western blotting was performed for wtEGFR and beta-actin. (D) EGFRvIII promotes cellular migration in vitro. Mock, wtEGFR, or EGFRvIII-transduced LN229 cells were suspended in upper chamber. After 6 hours of incubation in a 37°C incubator, the cells that migrated to the lower wells were observed fluorescently and counted the cell number. The data shown are the means ± SEM (n=4). Significant difference is shown: *** $p<0.001$.

Figure S2

Inhibition of Angptl4 mRNA expression by treatment with MAPK signal inhibitors. LN229-vIII cells were treated with inhibitors of various signaling pathways for 24 h. Total RNA was extracted and reverse- transcribed, followed by real-time PCR analysis. The following signaling inhibitors were used: U0126 (MEK inhibitor), SP600125 (JNK inhibitor), SB239063 (p38 inhibitor), LY294002 (PI3K inhibitor), AG490 (JAK kinase inhibitor), gefitinib (EGFR tyrosine kinase inhibitor), PD98059 (MEK1 inhibitor), FR180204 (ATP competitor of ERK1/2), ERK inhibitor III (Elk-1 and RSK inhibitor), and SL0101 (RSK inhibitor). The data shown are the means ± SEM (n=4). Significant differences are shown: * $p<0.05$, *** $p<0.001$.

References

1. Rae JM, Scheys JO, Clark KM, Chadwick RB, Kiefer MC, Lippman ME: **EGFR and EGFRvIII expression in primary breast cancer and cell lines.** *Breast Cancer Res Treat* 2004, **87:**87-95.
2. Fuse C, Ishida Y, Hikita T, Asai T, Oku N: **Junctional adhesion molecule-C promotes metastatic potential of HT1080 human fibrosarcoma.** *J Biol Chem* 2007, **282:**8276-8283.
