# Supplementary material for: Epidermal growth factor receptor variant type III markedly accelerates angiogenesis and tumor growth via inducing c-myc mediated angiopoietin-like 4 expression in malignant glioma
Source: Mol Cancer. 2013 Apr 25;12:31. doi: 10.1186/1476-4598-12-31 (PMC3641008; doi:10.1186/1476-4598-12-31)
Supplement: Additional file 3: Table S1 — Real time PCR analysis of angiogenesis-related genes in LN229-vIII cells. [file 1476-4598-12-31-S3.pdf]

Table S1. Real time PCR analysis of angiogenesis-related genes in LN229-vIII cells

Gene symbol	Mock	LN229-WT	LN229-vIII	Gene symbol	Mock	LN229-WT	LN229-vIII
ADAMTS1	1.00	0.70	0.58	IL12A	1.00	0.58	0.78
AMOT	1.00	0.87	1.14	ITGA4	1.00	0.70	0.63
ANGPT1	1.00	1.53	2.66	ITGAV	1.00	0.74	0.78
ANGPTL2	1.00	0.84	0.98	ITGB3	1.00	1.08	2.81
ANGPTL4	1.00	0.89	4.49	KIT	1.00	0.75	N.D.
CD44	1.00	0.74	0.68	MDK	1.00	0.85	0.70
COL15A1	1.00	1.67	0.34	MMP2	1.00	1.16	1.36
COL18A1	1.00	0.97	0.98	NRP1	1.00	0.85	0.96
COL4A1	1.00	0.73	0.40	NRP2	1.00	1.12	1.26
COL4A2	1.00	0.64	0.35	PDGFB	1.00	1.07	1.07
CTGF	1.00	0.56	0.56	PDGFRA	1.00	2.41	2.67
EDIL3	1.00	0.86	0.54	PDGFRB	1.00	2.10	0.47
ENPP2	1.00	0.93	1.05	PROX1	1.00	2.01	1.52
EPHB2	1.00	0.97	0.92	PTN	1.00	0.98	0.58
F2	1.00	0.90	N.D.	SERPINB5	1.00	0.87	3.66
FGF1	1.00	0.78	0.55	SERPINF1	1.00	0.59	0.77
FGF2	1.00	0.62	0.83	TGFA	1.00	1.15	1.45
FIGF	1.00	0.82	1.67	TGFB1	1.00	0.82	0.54
FLT1	1.00	0.83	0.52	THBS1	1.00	0.57	0.53
FN1	1.00	0.71	0.52	THBS2	1.00	1.30	3.15
FOXC2	1.00	0.71	0.23	TIE1	1.00	1.56	0.55
FST	1.00	0.53	0.34	TIMP2	1.00	1.03	1.06
GAPDH	1.00	0.78	0.97	TIMP3	1.00	1.31	1.51
GRN	1.00	0.91	0.92	TYMP	1.00	0.77	0.52
GUSB	1.00	0.85	0.78	VASH1	1.00	0.86	0.56
HEY1	1.00	1.49	2.38	VEGFA	1.00	0.69	1.43
HPRT1	1.00	0.79	0.72	VEGFB	1.00	0.96	0.93
HSPG2	1.00	0.76	0.44	VEGFC	1.00	0.62	1.11
IL8	1.00	0.25	1.29				

The data are shown as the mean (n=3)
N.D.: not detected
